# Supplementary material for: A Novel and Ubiquitous Marine Methylophage Provides Insights into Viral-Host Coevolution and Possible Host-Range Expansion in Streamlined Marine Heterotrophic Bacteria
Source: Appl Environ Microbiol. 2022 Mar 21;88(7):e00255-22. doi: 10.1128/aem.00255-22 (PMC9004378; doi:10.1128/aem.00255-22)
Supplement: Supplemental file 1 — Fig. S1 to S13 and legends of Tables S1 and S2. Download aem.00255-22-s0001.pdf, PDF file, 7.5 MB [file aem.00255-22-s0001.pdf]

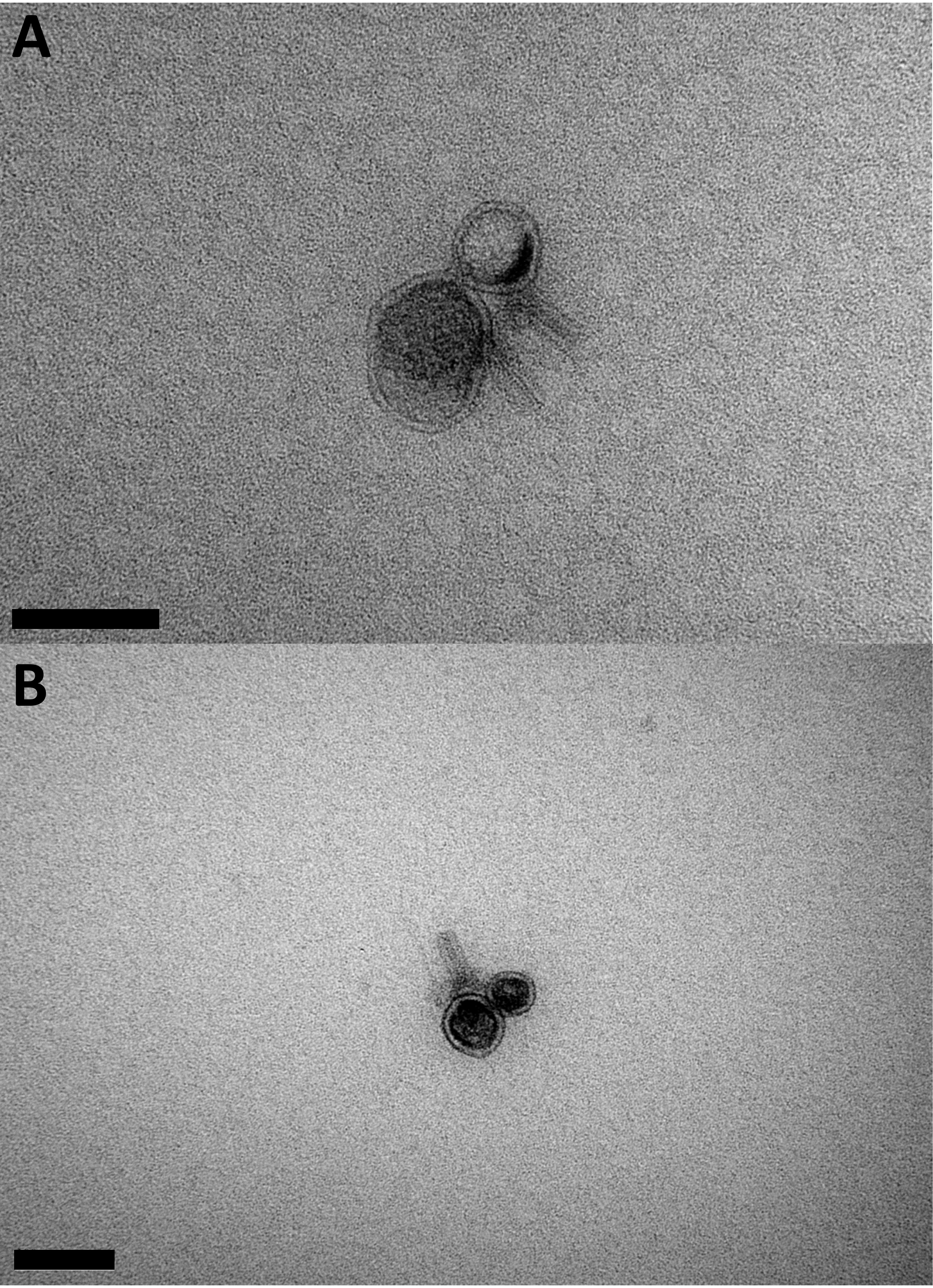
A
B

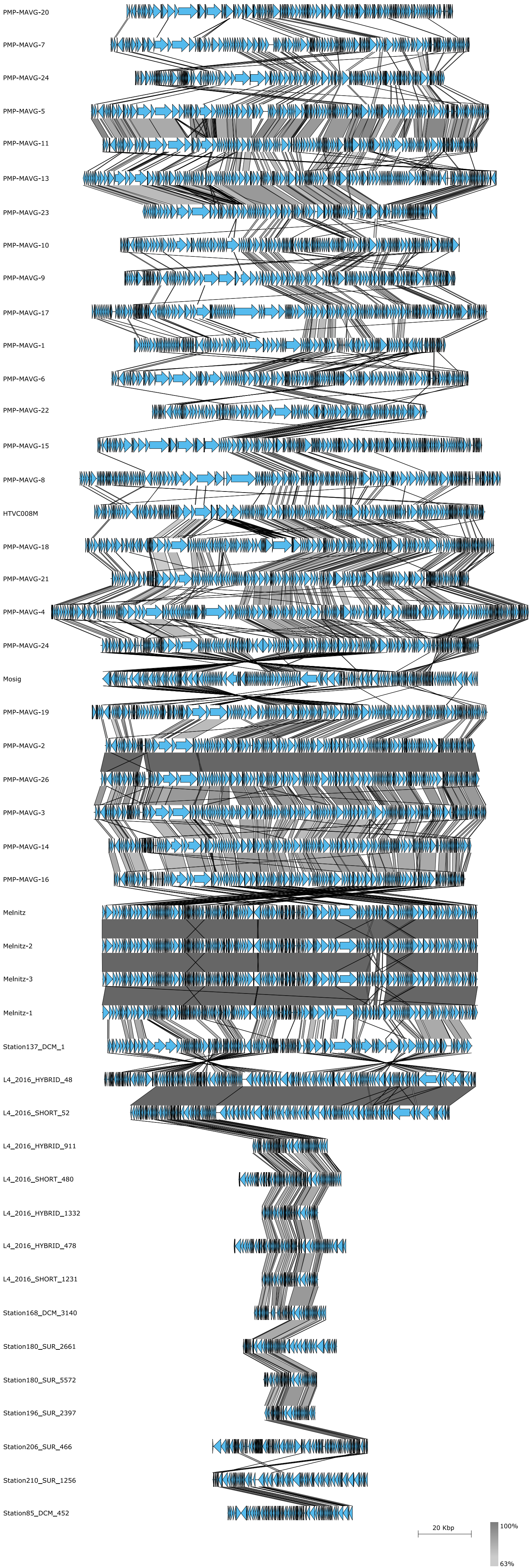
PMP-MAVG-20
PMP-MAVG-7
PMP-MAVG-24
PMP-MAVG-5
PMP-MAVG-11
PMP-MAVG-13
PMP-MAVG-23
PMP-MAVG-10
PMP-MAVG-9
PMP-MAVG-17
PMP-MAVG-1
PMP-MAVG-6
PMP-MAVG-22
PMP-MAVG-15
PMP-MAVG-8
HTVC008M
PMP-MAVG-18
PMP-MAVG-21
PMP-MAVG-4
PMP-MAVG-24
Mosig
PMP-MAVG-19
PMP-MAVG-2
PMP-MAVG-26
PMP-MAVG-3
PMP-MAVG-14
PMP-MAVG-16
Melnitz
Melnitz-2
Melnitz-3
Melnitz-1
Station137_DCM_1
L4_2016_HYBRID_48
L4_2016_SHORT_52
L4_2016_HYBRID_911
L4_2016_SHORT_480
L4_2016_HYBRID_1332
L4_2016_HYBRID_478
L4_2016_SHORT_1231
Station168_DCM_3140
Station180_SUR_2661
Station180_SUR_5572
Station196_SUR_2397
Station206_SUR_466
Station210_SUR_1256
Station85_DCM_452
20 Kbp
100%
63%

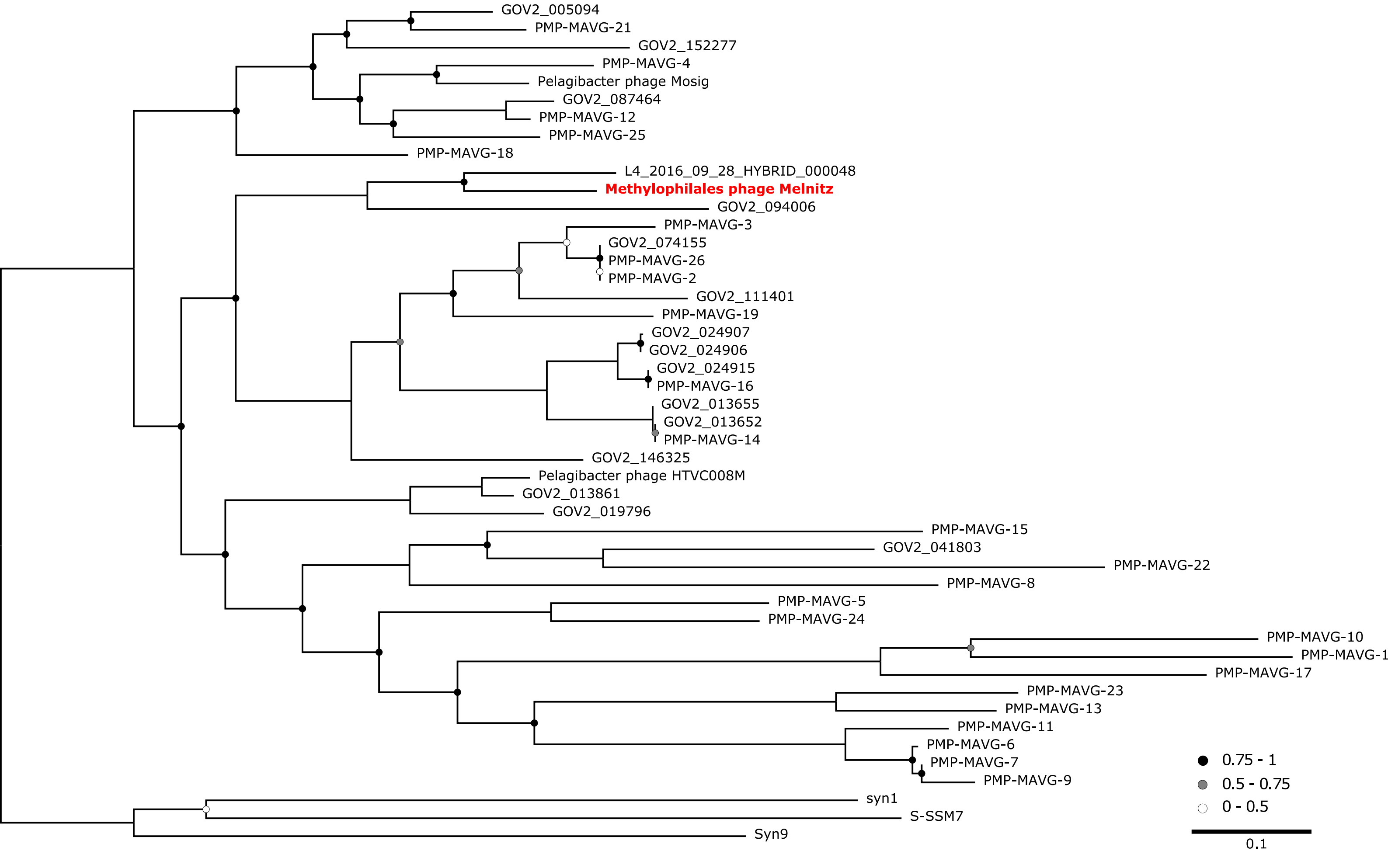
GOV2_005094
PMP-MAVG-21
GOV2_152277
PMP-MAVG-4
Pelagibacter phage Mosig
GOV2_087464
PMP-MAVG-12
PMP-MAVG-25
PMP-MAVG-18
L4_2016_09_28_HYBRID_000048
Methylophilales phage Melnitz
GOV2_094006
PMP-MAVG-3
GOV2_074155
PMP-MAVG-26
PMP-MAVG-2
GOV2_111401
PMP-MAVG-19
GOV2_024907
GOV2_024906
GOV2_024915
PMP-MAVG-16
GOV2_013655
GOV2_013652
PMP-MAVG-14
GOV2_146325
Pelagibacter phage HTVC008M
GOV2_013861
GOV2_019796
PMP-MAVG-15
GOV2_041803
PMP-MAVG-22
PMP-MAVG-8
PMP-MAVG-5
PMP-MAVG-24
PMP-MAVG-10
PMP-MAVG-1
PMP-MAVG-17
PMP-MAVG-23
PMP-MAVG-13
PMP-MAVG-11
PMP-MAVG-6
PMP-MAVG-7
PMP-MAVG-9
syn1
S-SSM7
Syn9
0.75 - 1
0.5 - 0.75
0 - 0.5
0.1

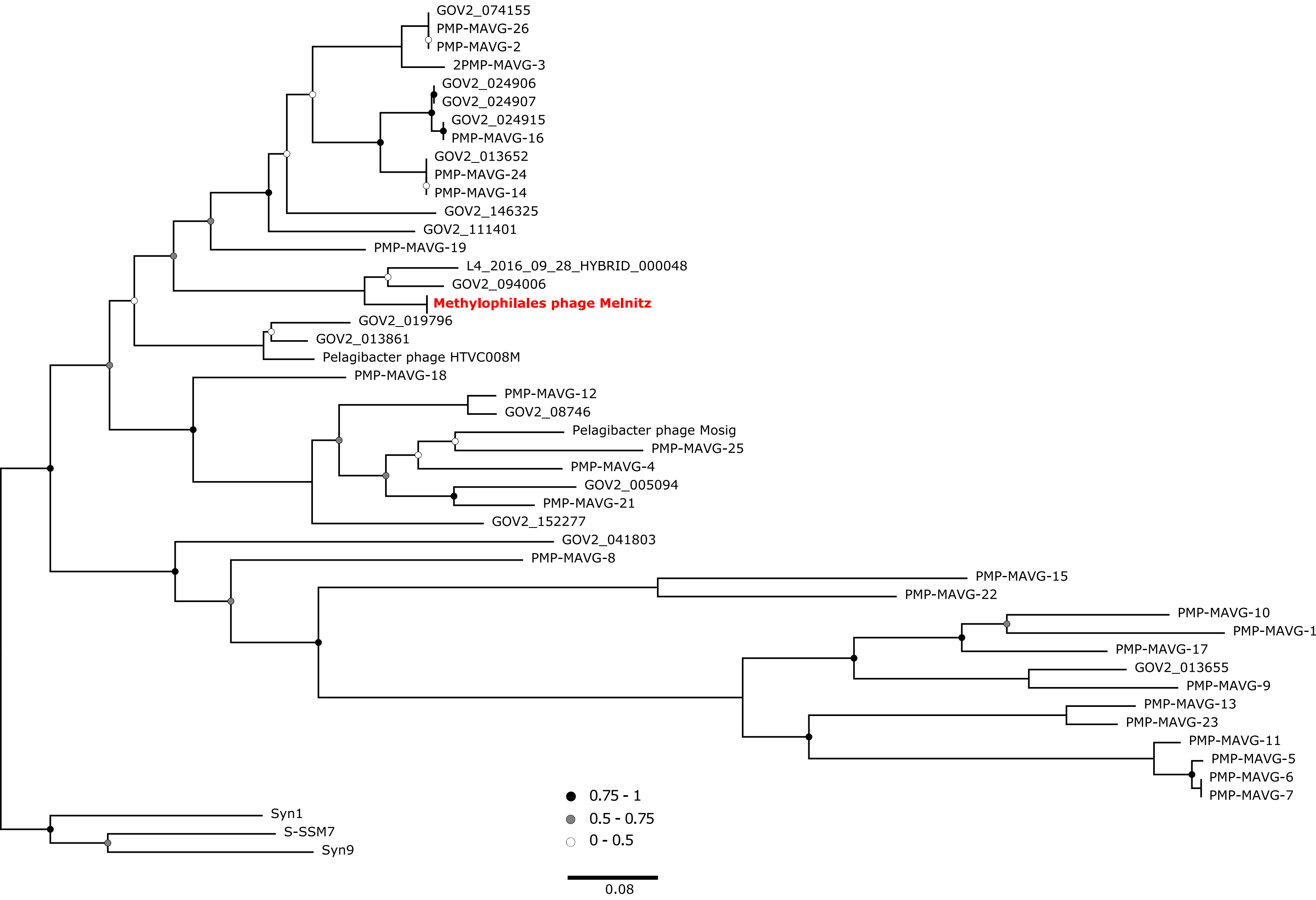

GOV2_074155
PMP-MAVG-26
PMP-MAVG-2
2PMP-MAVG-3
GOV2_024906
GOV2_024907
GOV2_024915
PMP-MAVG-16
GOV2_013652
PMP-MAVG-24
PMP-MAVG-14
GOV2_146325
GOV2_111401
PMP-MAVG-19
L4_2016_09_28_HYBRID_000048
GOV2_094006
Methylophilales phage Melnitz
GOV2_019796
GOV2_013861
Pelagibacter phage HTVC008M
PMP-MAVG-18
PMP-MAVG-12
GOV2_08746
Pelagibacter phage Mosig
PMP-MAVG-25
PMP-MAVG-4
GOV2_005094
PMP-MAVG-21
GOV2_152277
GOV2_041803
PMP-MAVG-8
PMP-MAVG-15
PMP-MAVG-22
PMP-MAVG-10
PMP-MAVG-1
PMP-MAVG-17
GOV2_013655
PMP-MAVG-9
PMP-MAVG-13
PMP-MAVG-23
PMP-MAVG-11
PMP-MAVG-5
PMP-MAVG-6
PMP-MAVG-7
Syn1
S-SSM7
Syn9
0.75 - 1
0.5 - 0.75
0 - 0.5
0.08

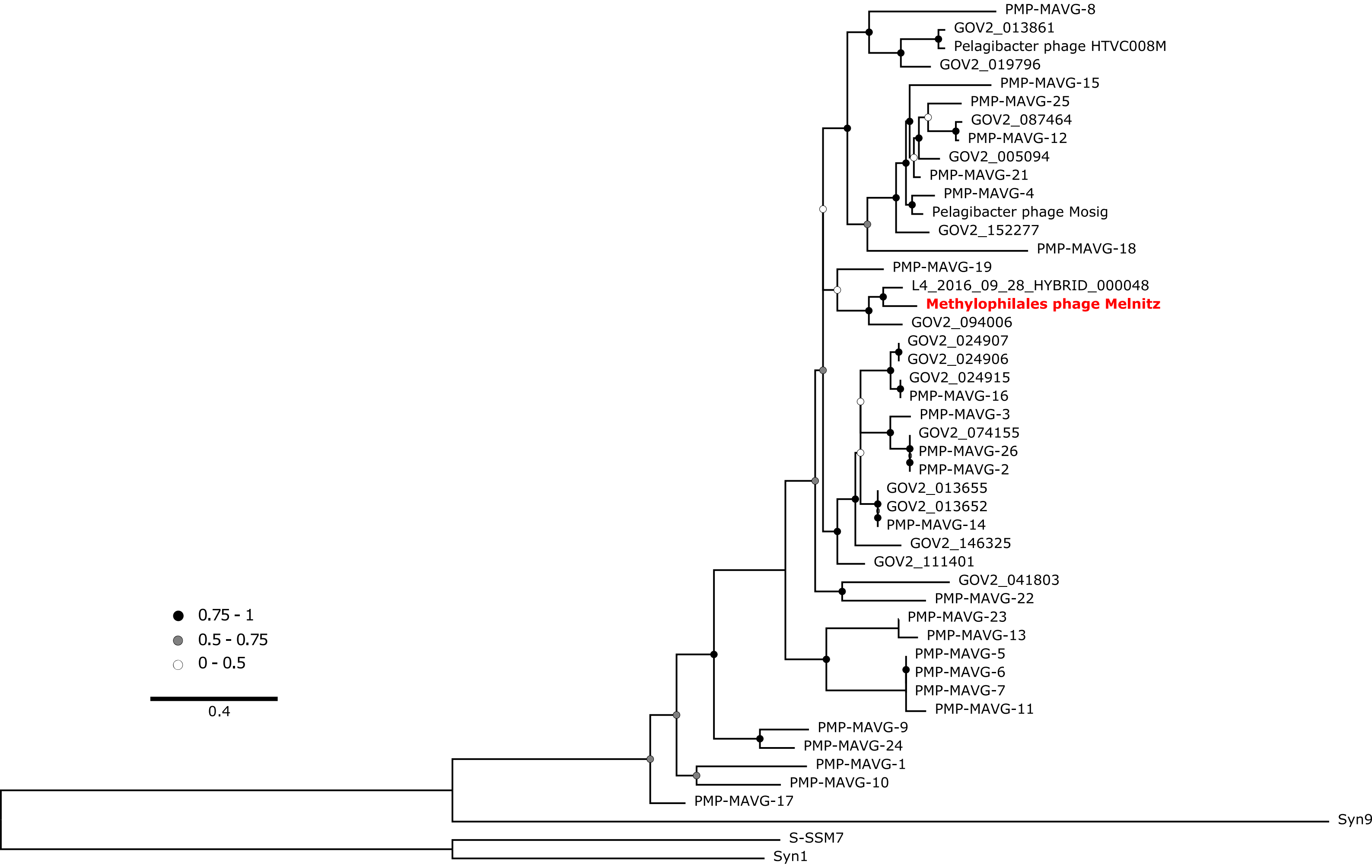

PMP-MAVG-8
GOV2_013861
Pelagibacter phage HTVC008M
GOV2_019796
PMP-MAVG-15
PMP-MAVG-25
GOV2_087464
PMP-MAVG-12
GOV2_005094
PMP-MAVG-21
PMP-MAVG-4
Pelagibacter phage Mosig
GOV2_152277
PMP-MAVG-18
PMP-MAVG-19
L4_2016_09_28_HYBRID_000048
Methylophilales phage Melnitz
GOV2_094006
GOV2_024907
GOV2_024906
GOV2_024915
PMP-MAVG-16
PMP-MAVG-3
GOV2_074155
PMP-MAVG-26
PMP-MAVG-2
GOV2_013655
GOV2_013652
PMP-MAVG-14
GOV2_146325
GOV2_111401
GOV2_041803
PMP-MAVG-22
PMP-MAVG-23
PMP-MAVG-13
PMP-MAVG-5
PMP-MAVG-6
PMP-MAVG-7
PMP-MAVG-11
PMP-MAVG-9
PMP-MAVG-24
PMP-MAVG-1
PMP-MAVG-10
PMP-MAVG-17
Syn9
S-SSM7
Syn1
0.75 - 1
0.5 - 0.75
0 - 0.5
0.4

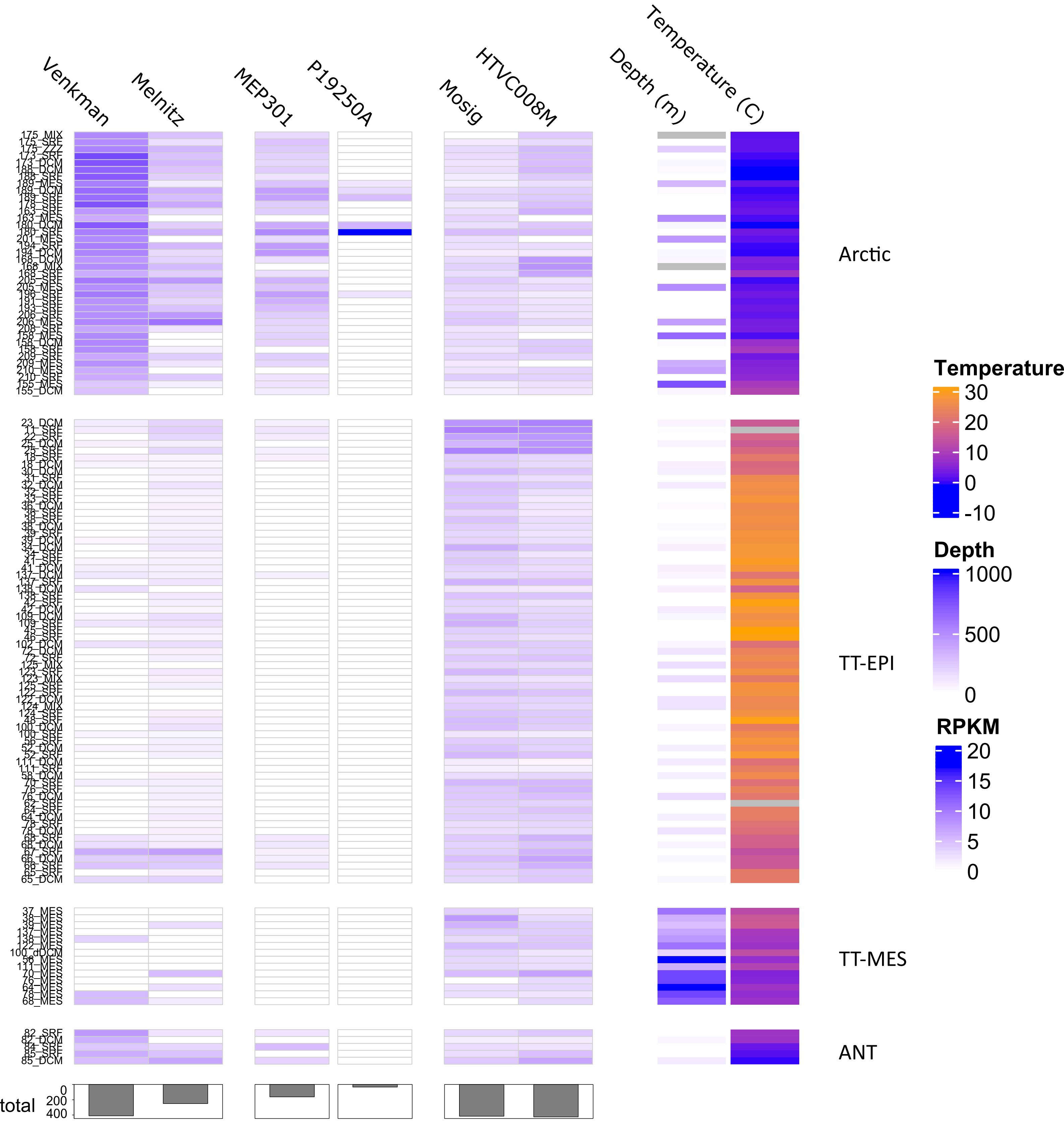

Venkman
Melnitz
MEP301
P19250A
Mosig
HTVC008M
Depth (m)
Temperature (C)
Arctic
TT-EPI
TT-MES
ANT
total
0
200
400
Temperature
30
20
10
0
-10
Depth
1000
500
0
RPKM
20
15
10
5
0

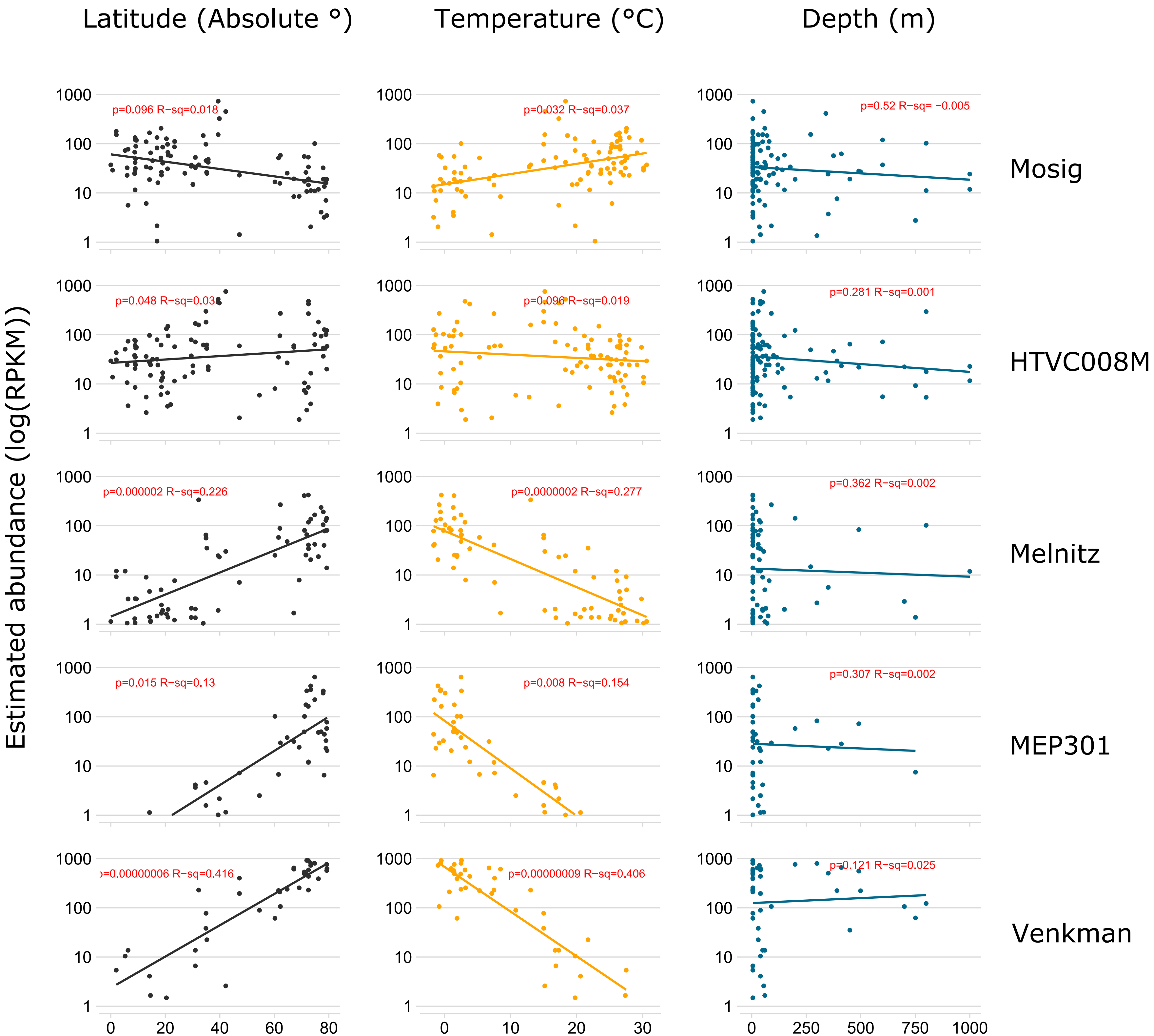

Latitude (Absolute °)
Temperature (°C)
Depth (m)
Estimated abundance (log(RPKM))
Mosig
HTVC008M
Melnitz
MEP301
Venkman
p=0.096 R−sq=0.018
p=0.032 R−sq=0.037
p=0.52 R−sq= −0.005
p=0.048 R−sq=0.03
p=0.096 R−sq=0.019
p=0.281 R−sq=0.001
p=0.000002 R−sq=0.226
p=0.0000002 R−sq=0.277
p=0.362 R−sq=0.002
p=0.015 R−sq=0.13
p=0.008 R−sq=0.154
p=0.307 R−sq=0.002
=0.00000006 R−sq=0.416
p=0.00000009 R−sq=0.406
p=0.121 R−sq=0.025
1000
100
10
1
0
20
40
60
80
0
10
20
30
0
250
500
750
1000

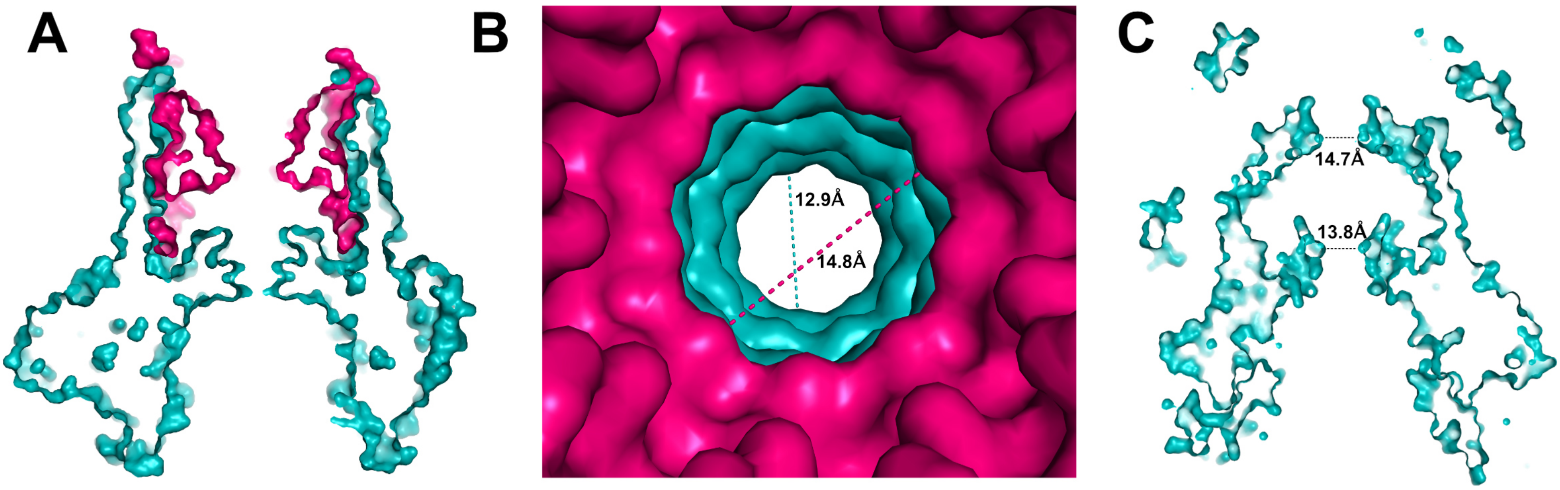
A
B
12.9Å
14.8Å
C
14.7Å
13.8Å

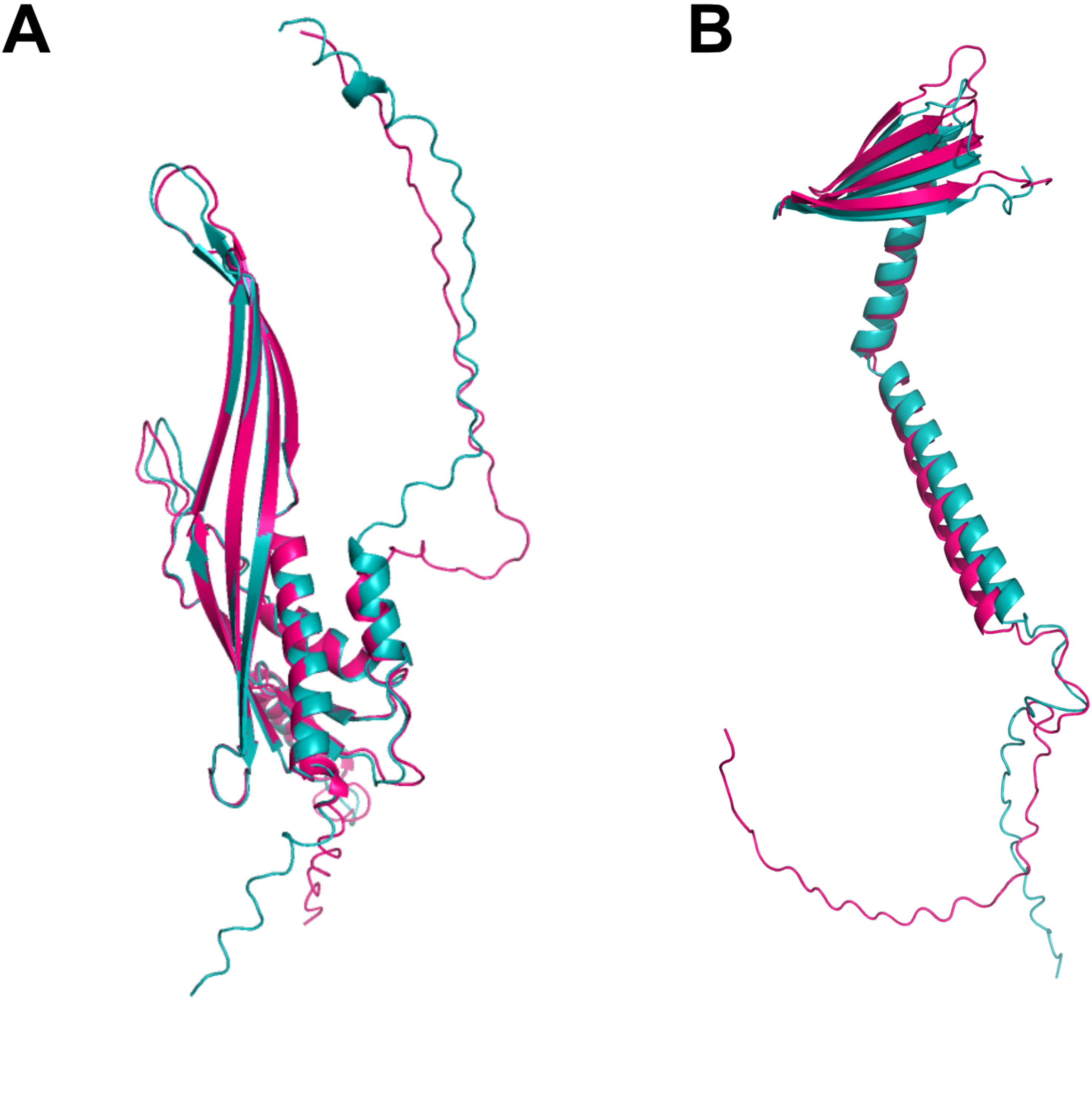
A
B

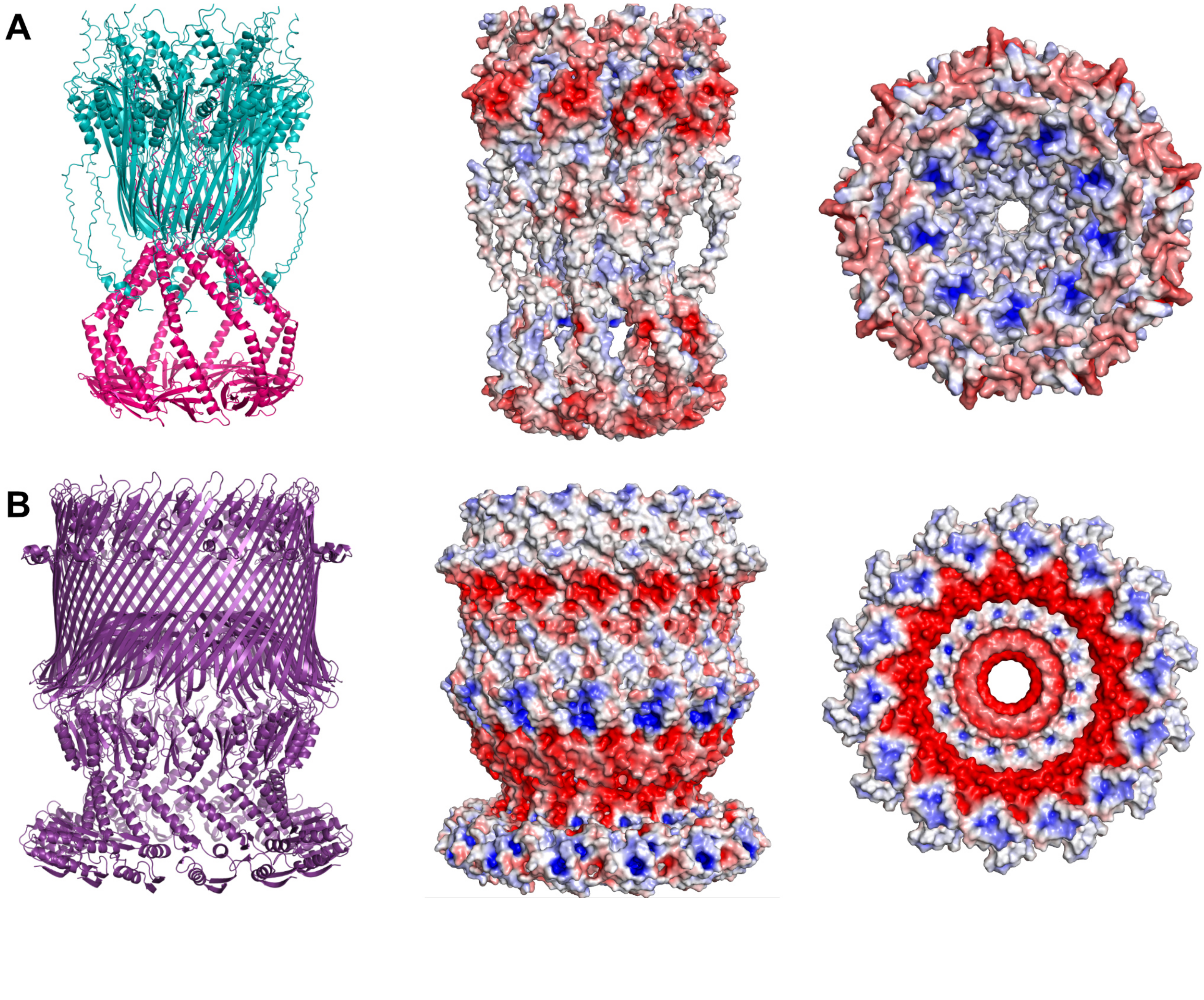
A
B

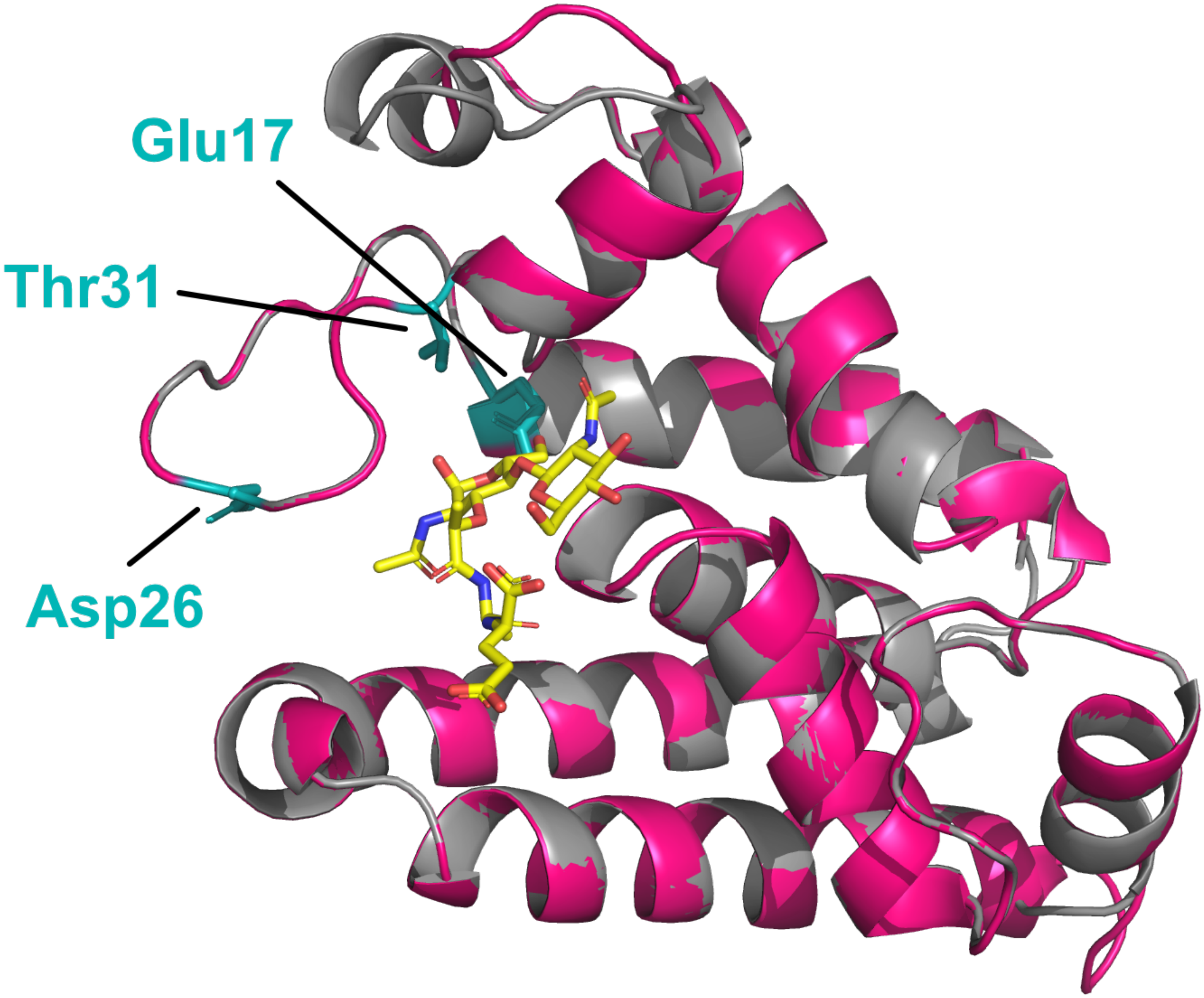

Glu17
Thr31
Asp26

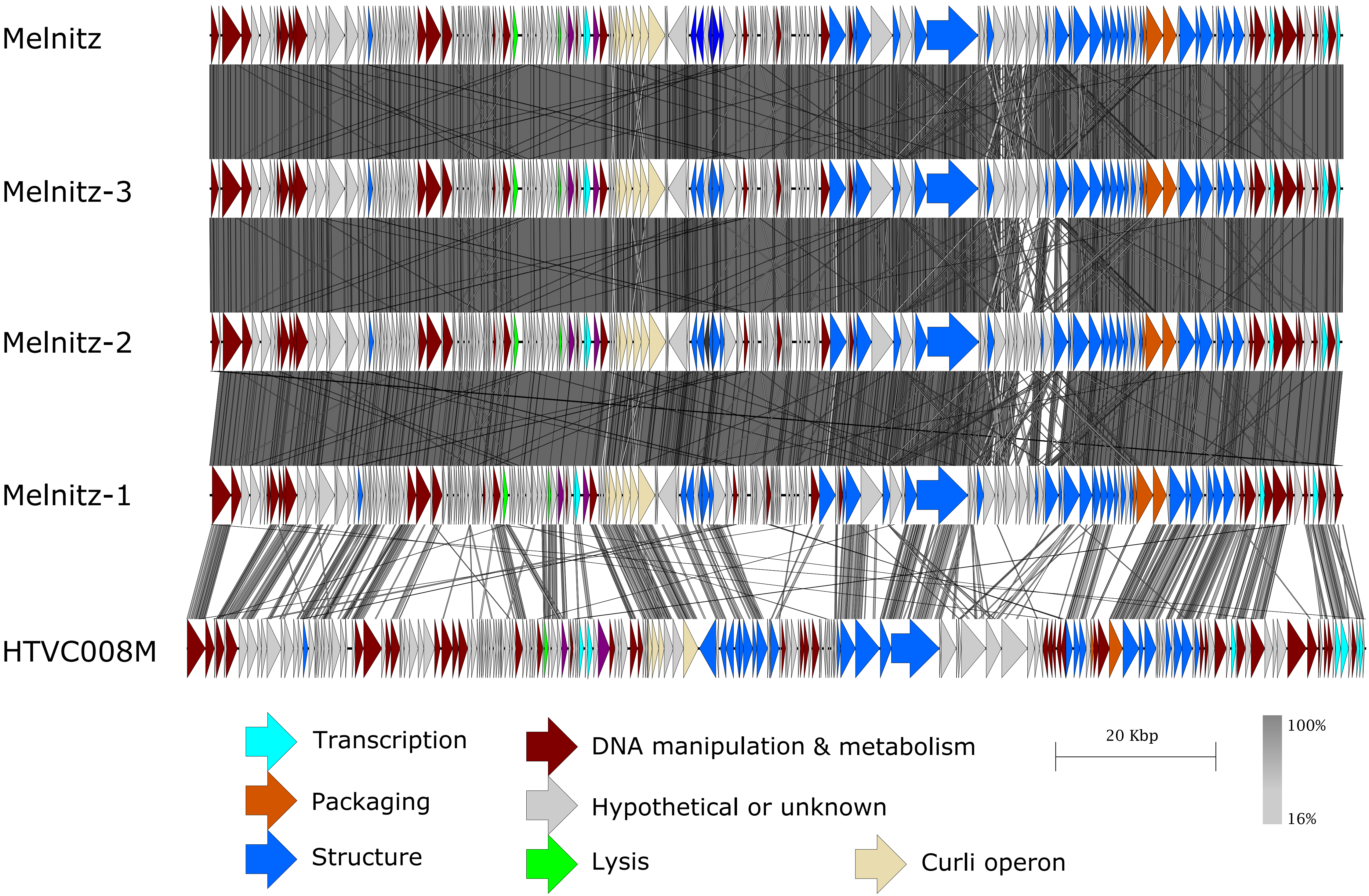
Melnitz
Melnitz-3
Melnitz-2
Melnitz-1
HTVC008M
Transcription
Packaging
Structure
DNA manipulation & metabolism
Hypothetical or unknown
Lysis
Curli operon
20 Kbp
100%
16%

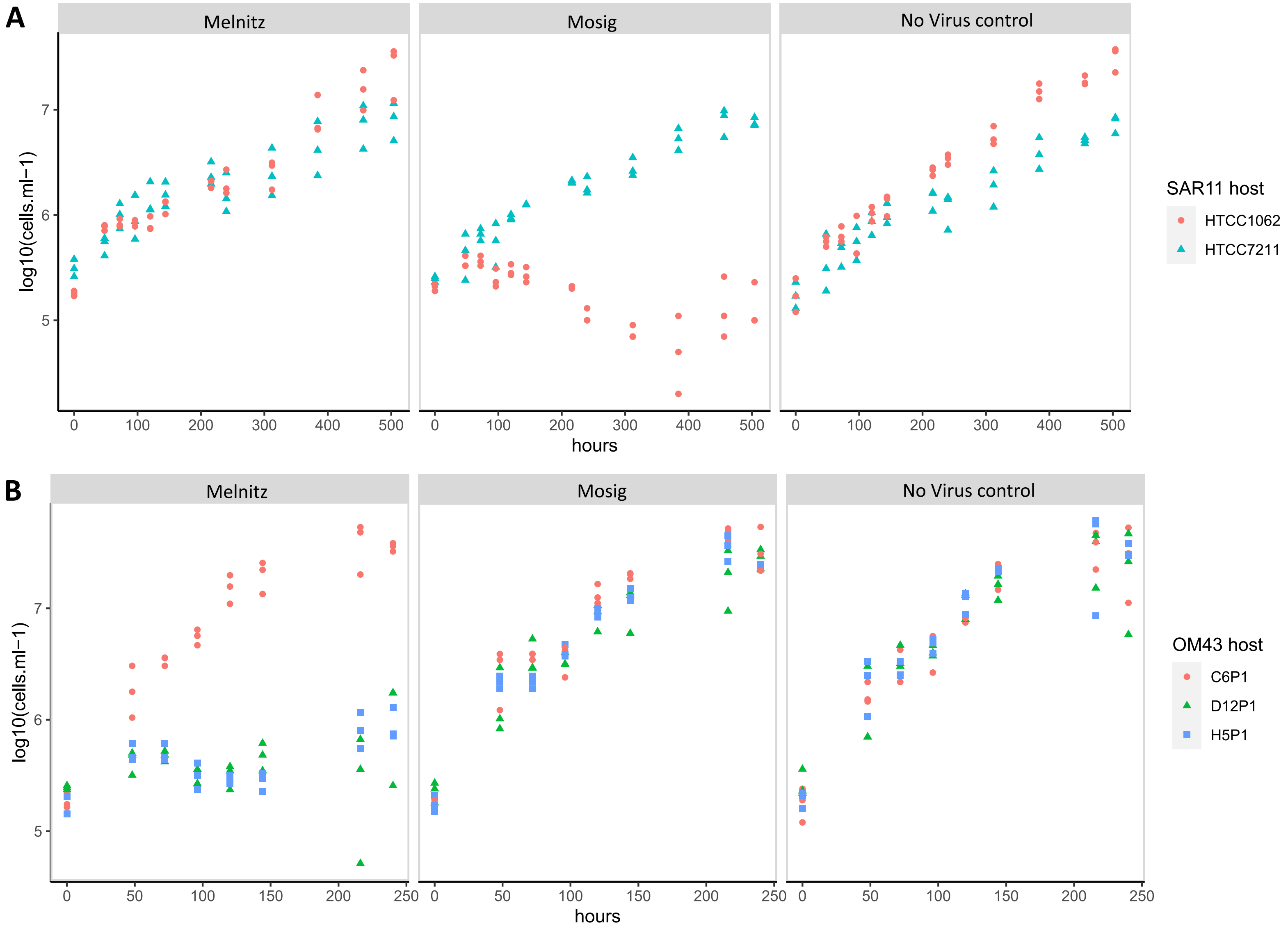

A
Melnitz
Mosig
No Virus control
log10(cells.ml−1)
hours
SAR11 host
HTCC1062
HTCC7211
B
Melnitz
Mosig
No Virus control
log10(cells.ml−1)
hours
OM43 host
C6P1
D12P1
H5P1

Supplementary Figure 1. TEM images of phage Melnitz virions. Bar indicates 100 nm.

Supplementary Figure 2. EasyFig visualisation of full length BLASTn alignments of MAVGs, environmental contigs and viral isolates related to Melnitz

Supplementary Figure 3. Neighbour-joining maximum-likelihood tree (100 bootstraps) of a tail sheath encoding gene found in Melnitz related myophages. Branch support values <1 are indicated by circle colours on the tree. The cyanophage branch was used to root the tree.

Supplementary Figure 4. Neighbour-joining maximum-likelihood tree (100 bootstraps) of the *TerL* gene found in Melnitz related myophages. Branch support values <1 are indicated by circle colours on the tree. The cyanophage branch was used to root the tree.

Supplementary Figure 5. Neighbour-joining maximum-likelihood tree (100 bootstraps) of a capsid scaffolding gene found in Melnitz related myophages. Branch support values <1 are indicated by circle colours on the tree. The cyanophage branch was used to root the tree.

Supplementary Figure 6. Global abundance of Melnitz. Reads recruited per kilobase of contigs per million reads (RPKM) of GOV2 viromes against phages infecting OM43 and LD28 as well as known *Pelagibacter* myophages. Samples are organized by ecological zone: Arctic, TT-EPI temperate-tropical epipelagic, TT-MES temperate-tropical mesopelagic, ANT Antarctic.

Supplementary Figure 7. Scatterplots of linear regression analyses using estimated abundances (RPKM) based on GOV2 virome read recruitment of the *Methylophilales* phages Melnitz, MEP301 and Venkman as well as *Pelagibacterales* phages Mosig and HTVC008M against GOV2 metadata (Latitude in absolute °, Temperature in °C, depth in metres).

Supplementary Figure 8. Cross sectional structure of CsgGF. **A** Clipped predicted surface model of internal structure of CsgGF in *E. coli* showing CsgG (teal) and CsgF (pink) showing the internal channel structure. This structure comprises a series of narrowing pores. **B** with CsgF creating a pore 14.8Å in diameter and CsgG creating a smallest pore 12.9Å in diameter. **C** Clipped predicted surface model of internal structure of Melnitz-encoded CsgG comprises two channels of similar size to those of the *E. coli* CsgGF complex.

Supplementary Figure 9. Structural comparison between **A** CsgG encoded by Melnitz (teal) and HTVC008M (pink); **B** CsgF encoded by Melnitz (teal) and HTVC008M (pink).

Supplementary Figure 10. **A** Predicted structure of Melnitz CsgF in an inverted orientation compared to **B** structure of secretin PilQ (6W6M) from *Vibrio cholerae*. Left to right - cartoon model; electrostatic potential (red = -ve, blue=+ve); top-down view through the barrel.

Supplementary Figure 11. SwissModel alignment of putative endolysin structure encoded by Melnitz gp67 (hot pink), with a SagA autolysin encoded by Brucella abortus (PDB model 7dnp.1, grey). The conserved peptidoglycan binding site encoded by Glu17, Asp26 and Thr31 is highlighted (teal), with the peptidoglycan substrate (yellow stick representation).

Supplementary Figure 12. Full length genome alignment of all four Melnitz variants against the *Pelagibacter* myophage HTVC008M shows a hight level of conservation of unusual genetic features such as the Curli operon and similar gene synteny.

Supplementary Figure 13. Host range assessment of Melnitz. **A** Growth curve of SAR11 strains HTCC1062 and HTCC7211, treated with OM43 phage Melnitz and SAR11 phage Mosig. **B** Growth curves of OM43 strains C6P1, D12P1 and H5P1 treated with OM43 phage Melnitz and SAR11 phage Mosig

Supplementary Table 1. List of phage genomes from NCBI with identified tmRNA feature.

Supplementary Table 2. Sequence similarity of viral-host tRNA alignments. These values were used in the construction of Figure 6.
